# Implementing group CBT-Insomnia in mental health and addictions services in London: initial results

**DOI:** 10.1192/j.eurpsy.2025.2341

**Published:** 2025-08-26

**Authors:** N. Stanton, C. Penny, L. Sugrue, J. Fehler, M. Lei-Yee Fok, J. D. King

**Affiliations:** 1 Central and North West London NHS Foundation Trust, London, United Kingdom

## Abstract

**Introduction:**

Chronic insomnia is prevalent in secondary care mental health populations and is associated with emotional distress, interpersonal impairment and reduced quality of life. In addition, it contributes to the aetiology of major mental health conditions and substance use. CBT for insomnia (CBTi) is a well-established evidence-based treatment approach for chronic insomnia and is recommended as the first-line treatment for adults in the UK by National Institute for Health and Care Excellence (NICE). Despite this, CBTi is not accessible for most secondary care mental health patients and therefore few benefit from this intervention.

**Objectives:**

To examine the feasibility and effectiveness of a 6-week group CBTi programme for people using secondary care mental health and addictions services with chronic insomnia, using a case series design.

**Methods:**

Each participant underwent an initial screening assessment to evaluate their suitability for the programme, the nature and impact of their sleep problem, and exclude other causes of insomnia. Self-reported measures of insomnia, personality functioning and depression were examined pre- and post-intervention using the Pittsburgh Sleep Quality Index (PSQI), Level of Personality Functioning Scale (LPFS) and Patient Health Questionnaire-9 (PHQ-9) respectively. PSQI was also re-assessed at 3-months’ follow-up.

**Results:**

Of 42 people referred to the service (26 from mental health and 16 from addictions services), 25 attended baseline assessment, 19 started the group and 12 completed sessions. The most common primary diagnoses were Alcohol Use Disorder (n=8), Treatment Resistant Depression (n=5), Bipolar Affective Disorder (n=3) and Personality Disorder (n=3). There were 9 men and 16 women. The severity of sleep disturbance was high with a cohort average PSQI of 15.4 (s.d. 2.7, range 12-20). Additionally, the level of personality functioning was high (mean 31.0, s.d. 7.6 range 13-45) as well as depressive symptoms (PHQ-9 cohort mean 18.0, s.d. 5.5, range 7-26).

Among the completers, cohort mean PSQI score decreased from 14.1 to 12.0 (p=0.12). Of 10 patients with 3-month follow-up data, there was a relative reduction of 20.3% from baseline, to a cohort mean PSQI score of 11.5 (p=0.16). At 3-months other facets of sleep quality like total sleep time had improved in the cohort by 45 minutes, and onset latency reduced by 35 minutes. Post-group there were also reductions in cohort mean LPFS scores by a relative 10.3% (31.4 at baseline to 28.3 post-group, and in cohort mean PHQ-9 by 14.8% (16.4 to 13.8).

**Conclusions:**

Group CBTi is a potentially scalable and feasible intervention that effectively treats chronic insomnia, depression and personality dysfunction in secondary care mental health and addictions populations. Further research should focus on replicating these findings in larger cohorts, and examine factors associated with uptake and completion of CBTi.

**Disclosure of Interest:**

None Declared

